# Potential of Casein as a Carrier for Biologically Active Agents

**DOI:** 10.1007/s41061-017-0158-z

**Published:** 2017-07-15

**Authors:** Tomasz Konrad Głąb, Janusz Boratyński

**Affiliations:** 1 Department of Biomedical Sciences, Faculty of Mathematics and Natural Sciences, Jan Dlugosz University in Czestochowa, 13/15 Armii Krajowej Avenue, 42-200 Czestochowa, Poland; 2 Laboratory of Biomedical Chemistry “NeoLek”, Hirszfeld Institute of Immunology and Experimental Therapy, Polish Academy of Sciences, 12 Rudolf Weigl Street, 53-114 Wroclaw, Poland

**Keywords:** Casein, Milk proteins, Encapsulation, Release, Delivery, Carriers

## Abstract

Casein is the collective name for a family of milk proteins. In bovine milk, casein comprises four peptides: α_S1_, α_S2_, β, and κ, differing in their amino acid, phosphorus and carbohydrate content but similar in their amphiphilic character. Hydrophilic and hydrophobic regions of casein show block distribution in the protein chain. Casein peptides carry negative charge on their surface as a result of phosphorylation and tend to bind nanoclusters of amorphous calcium phosphate. Due to these properties, in suitable conditions, casein molecules agglomerate into spherical micelles. The high content of casein in milk (2.75 %) has made it one of the most popular proteins. Novel research techniques have improved understanding of its properties, opening up new applications. However, casein is not just a dietary protein. Its properties promise new and unexpected applications in science and the pharmaceutical and functional food industries. One example is an encapsulation of health-related substances in casein matrices. This review discusses gelation, coacervation, self-assembly and reassembly of casein peptides as means of encapsulation. We highlight information on encapsulation of health-related substances such as drugs and dietary supplements inside casein micro- and nanoparticles.

## Introduction

**Fig. 1:**
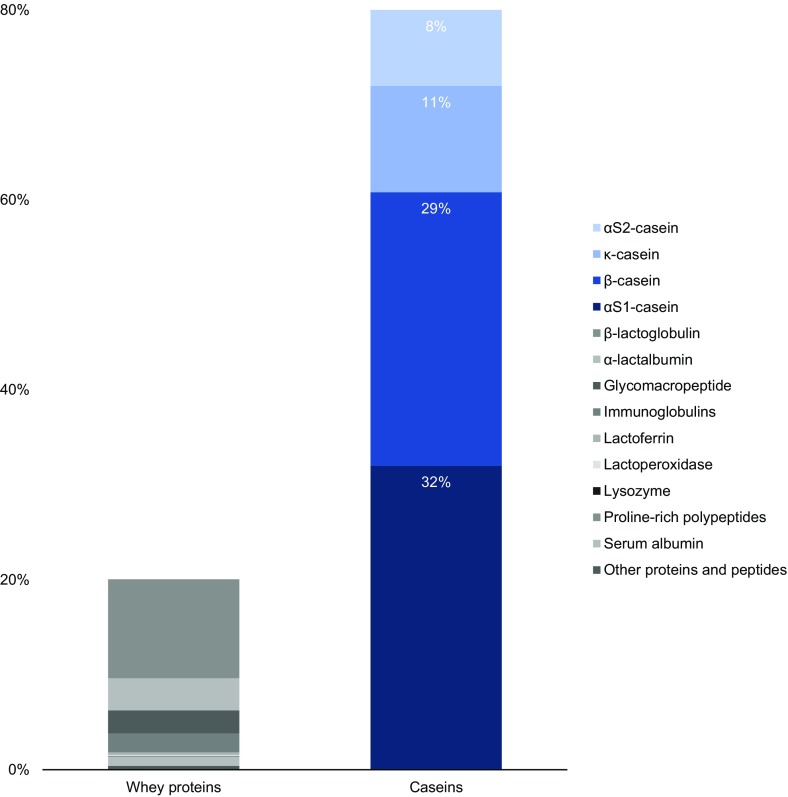
Standard protein content in bovine milk. Although whey proteins show greater diversity, caseins are more abundant. (Adapted and modified from Artym and Zimecki [3])

Casein (derived from the Latin *caseus* for ‘cheese’) [1] is the collective term for a family of secreted calcium (phosphate) binding phosphoproteins found in mammalian milk [2]. Caseins, in contrast to the second milk protein fraction, i.e. whey proteins, are insoluble and account for 80 % of total bovine milk proteins [3, 4], which translates to 2.75 % of total milk components (Fig. 1).

**Fig. 2:**
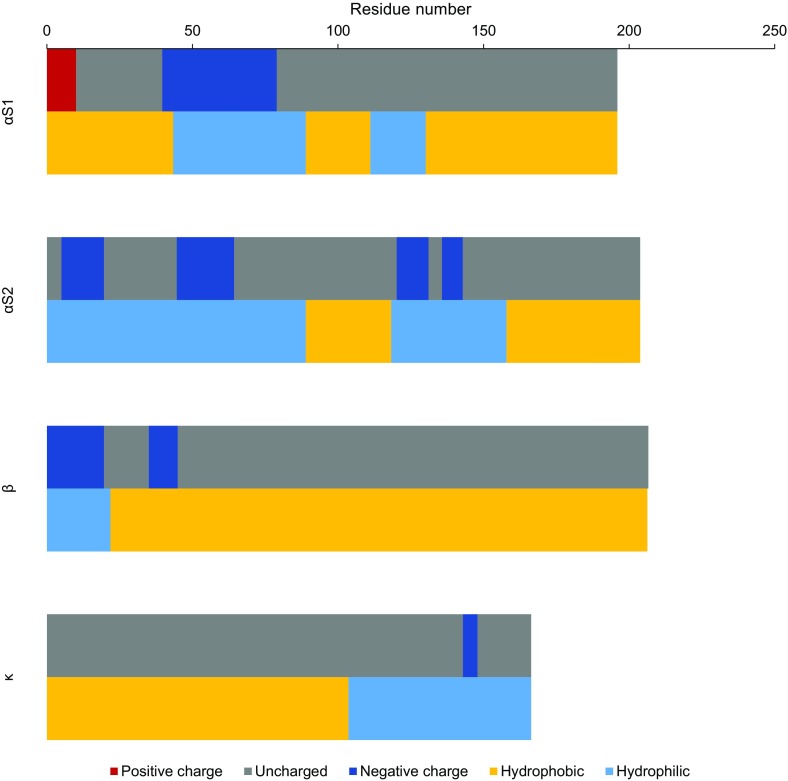
Schematic diagram of linear chain distribution of charged, hydrophilic and hydrophobic regions for the most common genetic types of caseins at the pH of milk (6.6). (Adapted and modified from Swaisgood and Kessler et al. [12, 13])

Eutherian milk contains products of at least three and at most five genes that encode casein peptides. Bovine milk contains products of four genes: α_S1_-, α_S2_-, β- and κ-casein, in assumed weight ratio of 4:1:4:1 [2, 5]. However, milk from various breeds of cow contains caseins in various proportions [6]. All casein peptides are amphiphilic (Fig. 2), but they differ in their amino acid, phosphorus and carbohydrate content (Table 1) [5, 7]. Only κ-casein, containing two cysteines, can form disulphide bonds [8, 9]. Secondary structures such as α-helices and β-sheets are not frequent, thus making caseins flexible, unfolded or random-coil peptides capable of creating intermolecular, e.g. electrostatic, hydrogen and hydrophobic, interactions [2, 10]. Therefore, in solution, caseins are present in a number of conformations that are most energy favourable [9]. Such conformations explain the low sensitivity of caseins to denaturation during, e.g. thermal treatment of milk [11].

**Table 1:** Differences in selected molecular properties of caseins

Fraction	Molecular weight (kDa)	Length of chain (aa)	Number of cysteine residues in chain	Number of phosphoserine residues in chain
α_S1_	24.5	214	1	9
α_S2_	26.0	222	3	10
β	25.1	224	1	4–5
κ	21.3	190	2	2

Data taken from UniProtKB database [16–19]

Caseins display activity similar to the small heat-shock proteins, in this case one casein molecule acting as a molecular chaperone towards the other casein molecule or other protein (e.g. whey protein), stabilising the target and preventing its unfavourable aggregation [2]. Therefore, under suitable conditions, casein peptides are present in the form of an amorphous, stable agglomerate known as a casein micelle (from the Latin *mica* for ‘crumb’ or ’morsel’) [11] with radius of 50–500 nm and mass of 10^3^–3 × 10^6^ kDa [9]. However, this broad distribution occurs in pooled milk, whereas the size of micelles is constant for a particular cow during milking, lactation and over a period of years [14]. The internal structure of the micelle is porous [15, 16]. Images obtained by cryo-transmission electron microscopy (cryo-TEM) show irregular channels, more than 5 nm in diameter, and inner cavities with diameter of 20–30 nm [15].

Caseins have capacity for binding phosphorus and calcium and proline- and glutamine-rich sequences, which are responsible for their intermolecular affinity [2, 4]. A typical casein micelle contains thousands of casein molecules, forming 94 % of the micelle. Most of them form thermodynamically stable complexes with amorphous calcium phosphate, which accounts for 6 % of the micelle [2, 5]. Amorphous calcium phosphate forms spherical nanoclusters with diameter of 3.5–5.0 nm, spaced ~18 nm apart [21]. Phosphate is bound to the protein by phosphoseryl residues [22]. An enzyme responsible for phosphorylation of caseins is Golgi kinase, known as Fam20C and not to be confused with casein kinase 1 and 2, named after a model substrate used for their identification [23].

**Fig. 3:**
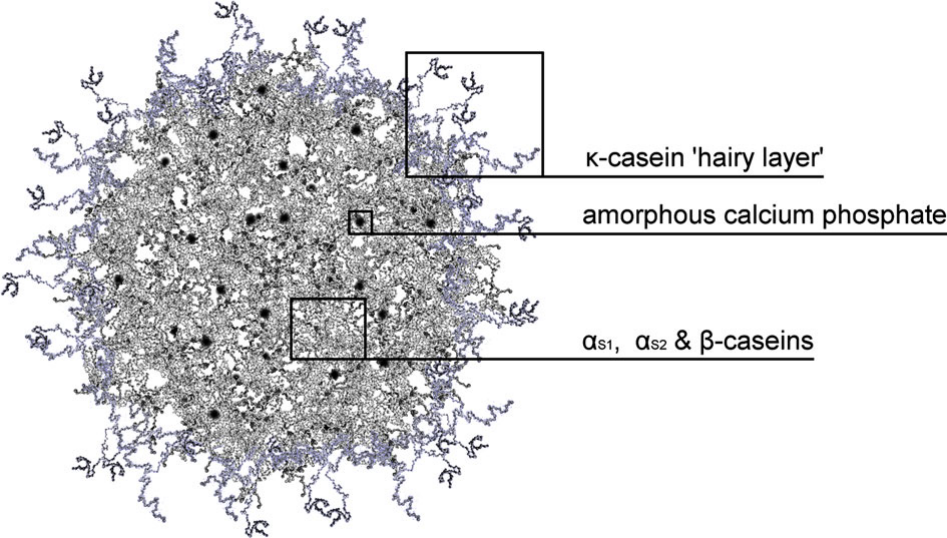
Original graphical representation of casein micelle cross-section according to the model proposed by Holt [27]. Artistic impression of casein micelle particle

In the structure of a casein micelle, peptides α_S1_, α_S2_, and β build up mostly in the inner part, while κ-casein forms the outer ‘hairy’ layer that stabilises the micelle sterically [5]. This stabilisation is possible because κ-casein has a glycosylated hydrophilic part that protrudes into the aqueous surrounding, known as glycomacropeptide [24, 25]. Interestingly, this ‘hairy’ layer is unevenly distributed and only partially covers the micellar surface [26] (Fig. 3).

The actual internal structure of a casein micelle remains uncertain, and various models have been constructed to describe it [14, 16]. Since casein is secreted by mammary epithelial cells [28], the distinctive structure of the micelles is an effect of evolution and plays an important biological role: calcium phosphate is sequestered in the core to avoid precipitation, and casein molecules aggregate around it to prevent formation of amyloid fibrils in mammary tissue [2], allowing mothers to feed neonates without ill consequences.

Casein micelles exhibit pH-dependent behaviour, tightening as the pH drops and swelling with increase of pH [29, 30]. The zeta potential measured for casein micelles was found to be −8 mV at neutral pH and close to 0 mV on decreasing the pH. Upon reaching the point where the micelles become unstable, it becomes hard to estimate the potential, but it is expected to be 0 at around pH 4.8 and have positive values at lower pH [31]. Thus, swelling of the micelles can be explained by a pH-driven increase of negative charge that results in stronger electrostatic repulsion between casein molecules, leading to loosening of the micellar structure and increase in size [30], while tightening can be explained by a decrease of charge with decreasing pH.

Lowering the pH below the isoelectric point of 4.6–4.8 causes aggregation and precipitation of micelles combined with release of calcium [5, 32, 33], indicating the great importance of cation availability for formation of casein micelles. In fact, studies on sodium and calcium caseinates [34, 35] as well as casein [36] show that different cations can influence the micelle structure in a complex manner.

**Fig. 4:**
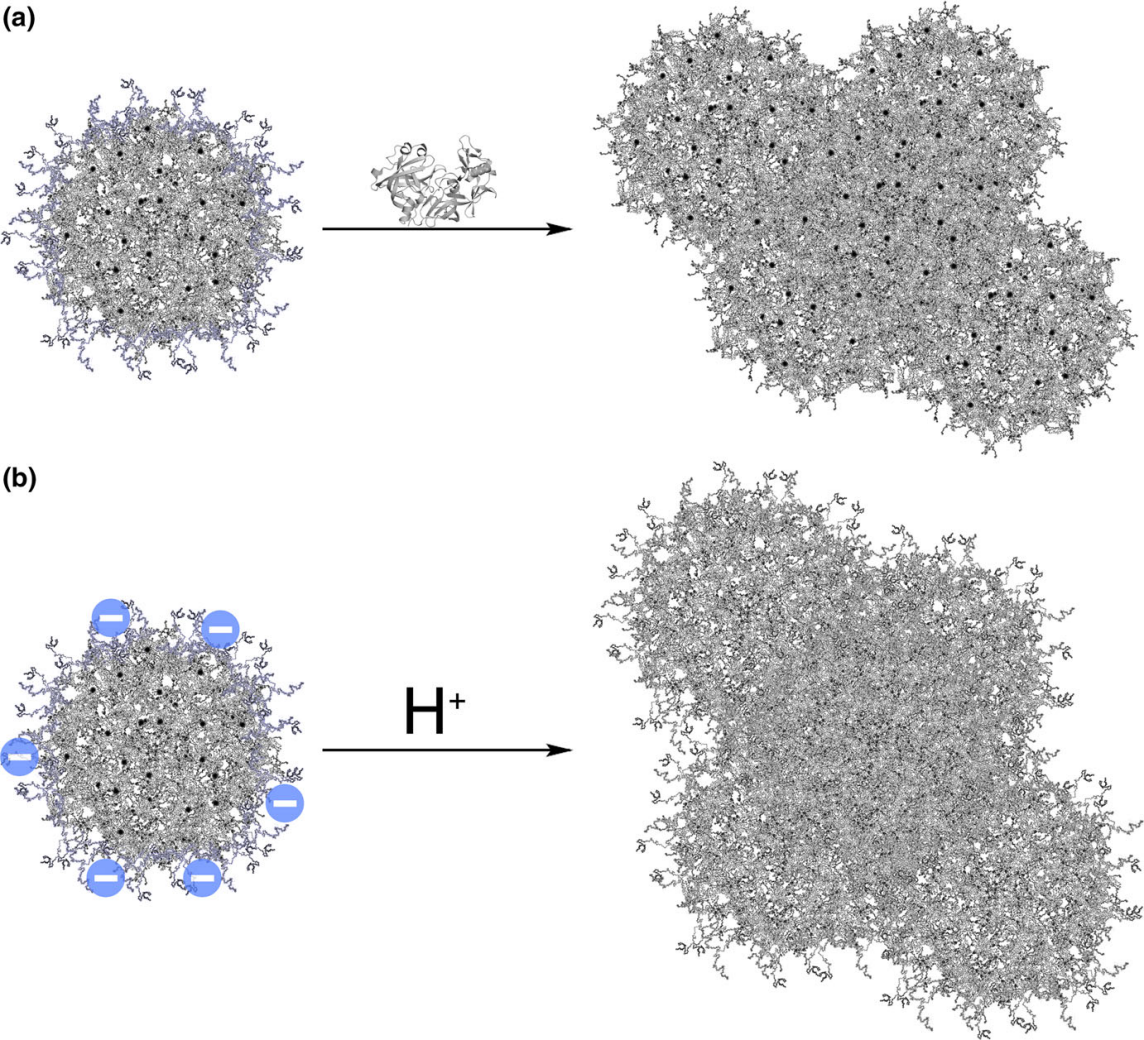
Methods commonly used for casein gelation. **a** Enzymatic method utilising chymosin, an enzyme found in rennet, for cleavage of hydrophilic κ-casein, leading to destabilisation of micelles and coagulation. **b** Lowering the pH to pI (4.6) using mineral (HCl) or organic (i.e. bacterial lactic) acid leads to neutralisation of surface charge and subsequent gelation

Casein has many advantages including low price and simple production. Industrial manufacture of casein involves a coagulation process that can be carried out by two means: enzymatic or acid gelation/precipitation (Fig. 4) as in cheese-making [10, 37]. Only acid casein can be easily resolubilised, which is achieved by converting it to salt, caseinate, using alkalis [10, 34].

Due to its simple processing, in addition to its obvious utilisation in the food industry, casein is also applied in other fields, including glues, plastics and textile fibres [10]. Additionally, due to its dietary role, casein is generally recognised as safe (GRAS), and offers biocompatibility, biodegradability and bioresorbability in oral administration. Those characteristics make casein a promising candidate for encapsulation matrix. The aim of this review is to present recent advances in encapsulation utilising casein.

## Casein Delivery Particles

As the pH is highly acidic in the human stomach but neutral in the duodenum [38], the pH-dependent behaviour of casein micelles can be beneficial for controlled release of substances administered orally. Additionally, caseins can penetrate the plasma membrane in an energy-independent fashion [39], which can enhance cellular uptake on oral administration. Furthermore, the unfolded structure of caseins makes them easily accessible for proteolysis [9], ensuring good release by proteolytic enzymes in the gastrointestinal tract. The amphiphilic nature of caseins allow micelles to exhibit natural affinity for hydrophobic substances [40] as well as hydrophilic macromolecules such as whey proteins and polysaccharides [41]. All of these features make casein a promising candidate for encapsulation matrix.

Casein delivery particles can be categorised based on their type and size. According to the International Union of Pure and Applied Chemistry (IUPAC) definition, particles can be divided into capsules with a solid shell and core space available to entrap substances, and spherical particles without a membrane or outer layer. In terms of size, particles with dimensions of 0.1–100 µm are granted the prefix ‘micro’, whereas particles in the range of 1–100 nm are prefixed ‘nano’. However, the size limit between ‘micro’ and ‘nano’ remains controversial [42].

Casein has been used as an encapsulation material for all the above-mentioned types of particles. Different substances have been encapsulated, possessing pharmaceutical, health-related and nutritional value. Even though approaches for encapsulation of nutraceuticals and nutrients may differ from methods used to encapsulate pharmaceuticals, the former are still interesting and may find pharmaceutical applications as well. In fact, many preparations initially tested for encapsulation of nutraceuticals were eventually tested for drugs.

### Microparticles

The first papers on use of casein for microencapsulation were published in the late 1980s [43, 44], and since then much work has been carried out. This review highlights the most recent research.

Casein exhibits outstanding gelation properties, which have been utilised together with emulsification for encapsulation of probiotic bacteria such as *Bifidobacterium lactis*, *Lactobacillus casei*, *Lactobacillus paracasei* and *Lactobacillus rhamnosus* [45–48].

**Fig. 5:**
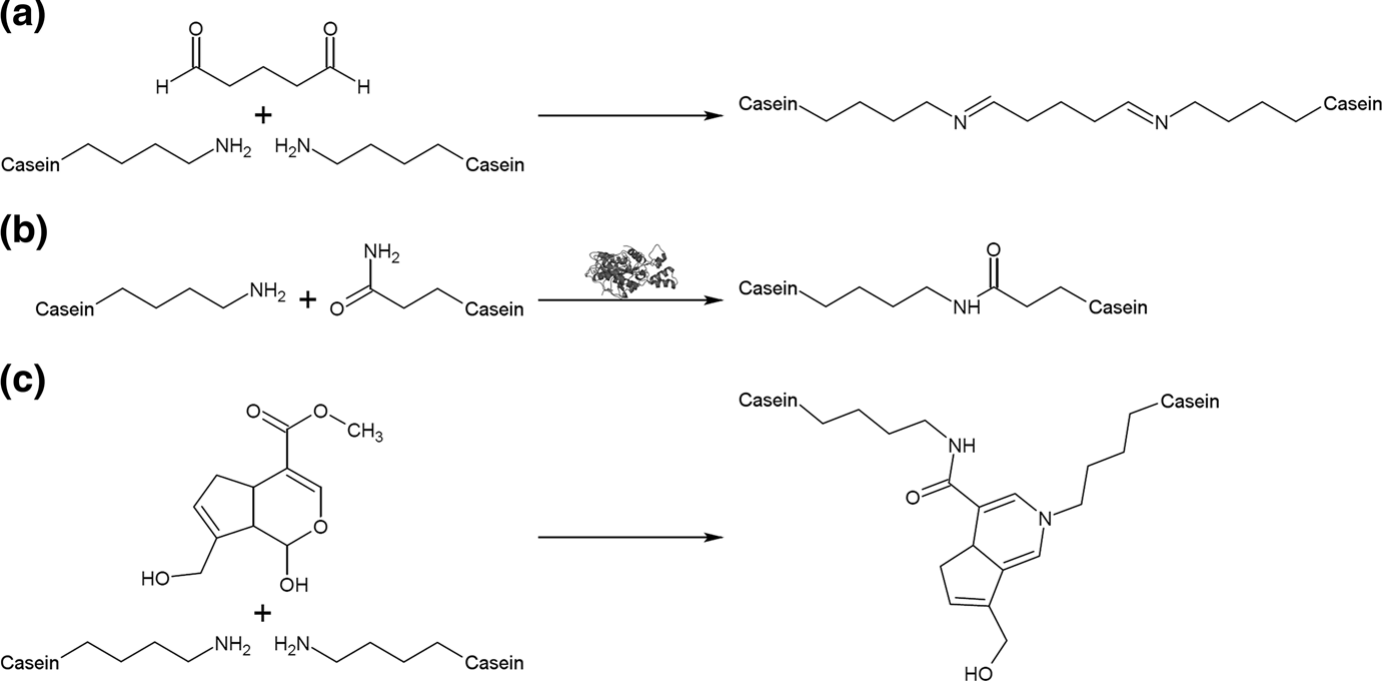
Schematic reactions for different agents used for cross-linking of casein. **a** Reaction of glutaraldehyde with ε-amine groups of protein yields a non-conjugated Schiff base as a result of nucleophilic attack on the aldehyde groups. Additionally, in alkaline solution, polymeric glutaraldehyde can form and react with proteins [49]. **b** Enzymatic acyl-transfer reaction between γ-carboxamide group of glutamine residue and ε-amino group of lysine residue facilitated by transglutaminase results in ε-(γ-glutamyl)lysine isopeptide bonds [50]. **c** Genipin cross-linking results from nucleophilic attack on genipin ring by primary amine group followed by nucleophilic substitution of ester group [51]

One of the methods used to achieve this is cross-linking the protein with transglutaminase (Fig. 5). Such cross-linking results in gelation of the casein and thus entrapment of bacterial cells [46, 47]. However, after freeze-drying and 3 months of storage in optimised conditions (4 °C and relative humidity of 11 %), significant loss of living cells was observed. This phenomenon is thought to be unavoidable, hence authors have proposed addition of protective substances [47].

Casein and whey proteins are other materials used for encapsulation of probiotic bacteria [45]. Micellar casein and/or whey proteins were mixed with probiotic cells, and rennet was added to induce gelation. Subsequently, the product was subjected to gastric digestion in simulated gastric fluid and monitored in situ using a particle size and shape analyser. A formulation consisting of denatured whey proteins and micellar casein gave the best results in terms of distribution breadth, elastic modulus, encapsulation rate, half-time of gastric digestion, particle size and survival rate.

Even though casein microparticles can resist low pH, they are readily digested by pepsin in vitro and offer insufficient protection to probiotic bacteria in vivo. To prevent this phenomenon, a fat coating was introduced, granting these particles resistance to pepsin digestion in vitro. However, when tested on mice, the particles were still easily digested, presumably through the action of gastric lipases [48]. Nonetheless, it is possible that other coating materials could be suitable for this task.

Recently, spray-drying instead of emulsification was also used for microencapsulation of probiotic bacteria [52]. Micellar casein was mixed with denatured whey proteins and chymosin. After enzymatic cleavage of κ-casein, *L. rhamnosus* lyophilisate was added and the mixture was spray-dried. The survival rate of bacterial cells after spray-drying was satisfactory, but the preparation was not tested in model gastric conditions. Nonetheless, the obtained microparticles exhibited interesting behaviour during reconstitution in water: at 8 °C, probiotic cells were released, while at 40 °C, gel formation caused entrapment. This finding offers perspectives for storage of probiotic cells.

Caseins were found to interact with polysaccharides, forming coacervate-type complexes [53–56]. Coacervation is triggered by adjusting the pH below the isoelectric point of casein. Such low pH is necessary as both casein and polysaccharides have negative charge in neutral pH, repelling each other. Shifting the pH to low values results in positive charge on the proteins, allowing them to interact with the negatively charged polysaccharide chains [55].

The coacervation method was reported to be suitable for encapsulation of volatile compounds [55, 56]. After mixing sodium caseinate or whey protein isolate with carboxymethyl cellulose, β-pinene was added and oil-in-water emulsions were prepared. Coacervation was conducted at pH 2.8. Higher protein-to-polysaccharide ratio was more beneficial through the formation of a network in which β-pinene could be entrapped, being more evident in the case of whey proteins, supposedly due to partial unfolding at low pH [55]. Addition of reticulating agents, cross-linking via hydrogen bonds, during coacervation gave contradictory results: tannic acid did not induce any significant change, whilst glycerol gave a 1.5- to 2-fold increase in encapsulation efficiency in the case of caseinate and above 2.2-fold in the case of whey protein isolate. The authors attributed these differences to the size of the compounds utilised, as the small glycerol molecules were able to fill the pores in the microcapsules sealing them, while tannic acid could cause disruption of the matrix during coacervation [56].

Jain et al. used gum tragacanth for coacervation with casein to encapsulate β-carotene [53] and lycopene [54]. Briefly, the bioactive compounds were dissolved in rice bran oil and emulsified with casein. Gum tragacanth was added to obtained oil-in-water emulsions, and the pH was adjusted from 10.5 to 2.0 to trigger coacervation. Complexes were then treated with genipin to cross-link the coating material. Despite the initial burst release attributed to adsorbed or externally encapsulated β-carotene, microcapsules generally exhibited a good release curve during in vitro release studies. Additionally, microcapsules retained good stability during 2 months of storage, especially at 4 °C [53]. The stability and residual action of lycopene-loaded capsules were shown to be significantly enhanced in comparison with lycopene in oil. Furthermore, twofold better bioavailability was observed in rats for microencapsulated lycopene [54].

In other work, casein was coacervated with pectin. Indomethacin was mixed with casein/pectin solution, and coacervation was triggered by slowly reducing the pH to 3.5 [57]. The same procedure was followed for acetaminophen (paracetamol). Some formulations used in this study were additionally cross-linked with glutaraldehyde. The obtained microparticles were able to prolong indomethacin release, and the interaction between casein and pectin did not prevent the enzymatic breakdown of pectin. However, cross-linking inhibited enzymatic digestion of pectin without prolonging release. The formulation was shown to be unsuitable for water-soluble drugs such as acetaminophen, which exhibited rapid release.

Casein was shown to act as an antioxidant in emulsions and microspheres [58]. Microspheres were prepared from sodium caseinate and pectin, emulsified with fish oil, followed by cross-linking with transglutaminase. The stability of microencapsulated fish oil was then compared with that of emulsions prepared with fish oil and casein or Tween 20. Specimens stabilised with caseinate oxidised slower than those stabilised with Tween 20, and interestingly no significant differences were shown between caseinate emulsions and caseinate microspheres.

Flaxseed oil and quillaja saponin were emulsified and mixed with sodium alginate and sodium caseinate. Then, using an encapsulation unit, droplets were injected into calcium chloride solution to facilitate gelation. Sodium caseinate was shown to effectively inhibit lipid oxidation during 50 days of storage at 55 °C [59].

Flaxseed oil was also microencapsulated in caseinate and whey protein concentrate-based microcapsules prepared by emulsification followed by spray-drying. After 6 months of storage at 35 °C, the microcapsules showed high oxidative stability. When tested in simulated gastric fluid and simulated intestinal fluid conditions, the caseinate microcapsules showed nearly twofold higher release than whey protein concentrate capsules [60].

### Nanoparticles

Many biofluids, including milk, contain phosphate and calcium in concentrations exceeding the limits of solubility that are stabilised by proteins [21]. Caseins fulfill this role through their natural ability to self-assemble into micelles in presence of calcium phosphate [61]. In some way, this phenomenon can be perceived as encapsulation invented by Nature itself. The concentration of casein monomers at which micelles appear, i.e. the critical micelle concentration (CMC), is considered to be 1.0 mg/ml [29], but various factors affect casein micellisation in different ways. Furthermore, hydrophobic regions of caseins can interact with other hydrophobic substances. These characteristics can be harnessed to create delivery nanoparticles.

**Fig. 6:**
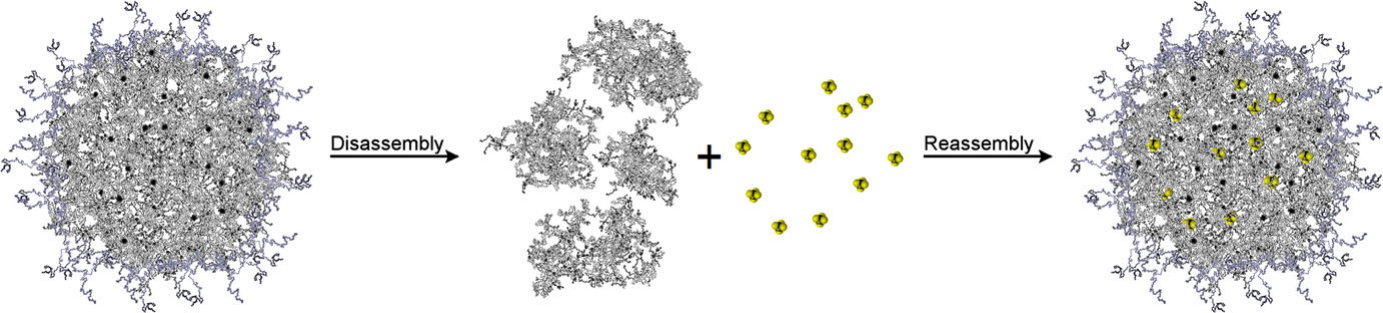
Graphical representation of general principle behind reassembled casein nanospheres. Briefly, the micellar structure is disrupted, lipophilic compounds dissolved in organic solvents bind to hydrophobic regions of casein peptides, then caseins are reassembled, creating new, substance-loaded nanoparticles

The first report treating casein micelles as nanocapsules was published by Semo et al. Those authors were able to incorporate fat-soluble vitamin D_2_ in hydrophobic regions of reconstituted casein micelles [40]. Moreover, two different papers concerning binding of lipophilic compounds to hydrophobic regions of caseins were published at roughly the same time, but the authors did not consider the obtained products to be capsules [62, 63]. This led to a series of other studies concerning encapsulation of hydrophobic compounds in reassembled casein micelles using different approaches (Table 2). The process of creating loaded reassembled casein micelles consists of three phases. The first step is disruption of the micellar structure, which is achieved by, inter alia, high-pressure treatment, ultrasound treatment or simply using caseinate already deprived of its micellar structure [40, 64, 65]. Then, lipophilic substances dissolved in organic solvents such as ethanol are added. Ethanol has a dissociative effect on casein, presumably due to increased solubility of caseins by reduction of phosphoseryl cross-linking and increased protein hydrophobicity [66]. In the next step, removal of disrupting agent and organic solvent allows hydrophobic substances to bind to lipophilic regions of caseins and the micelles to reassemble (Fig. 6). In the case of caseinate, an extra step is necessary to recreate the micellar structure: restoration of the mineral composition achieved by addition of certain salts [40].

**Table 2:** Techniques utilised for preparing loaded reassembled casein micelles

Category	Substance	Technique	References
Pharmaceuticals	Triclosan	High pressure homogenisation	[63]
	Flutamide	Spray-drying	[65] [66]
	Curcumin	Spray-drying	[67]
		pH-shifting	[68]
		High pressure treatment	[69]
Nutraceuticals	Vitamin D_2_	Restoration of mineral composition and ultra-high pressure homogenisation	[59]
		High pressure treatment	[70] [71]
	Vitamin D_3_	Restoration of mineral composition and ultra-high pressure homogenisation	[72] [73] [74]
	β-carotene	Solvent displacement	[60]
		Emulsification-evaporation	[61]
		Restoration of mineral composition and ultra-high pressure homogenisation	[75]
		Restoration of mineral composition and spray-drying	[76]
		High pressure homogenisation	[77]
		Restoration of mineral composition	[78]
	Vitamin B_9_	Coacervation and spray-drying	[79]
	ω-3 polyunsaturated fatty acids	Restoration of mineral composition and ultra-high pressure homogenisation	[80]
	Fucoxanthin	Restoration of mineral composition and spray-drying	[81]
Nutrients	Soybean oil	pH changes and ultrasound treatment	[62]
	Rapeseed oil		
	Fish oil

Recently, reassembled casein nanocapsules loaded with vitamin D_3_ were shown to provide bioavailability of vitamin D comparable to that in Tween 80 [74, 75] and successfully underwent clinical trials, showing bioavailability similar to that in fat [76]. Furthermore, different casein nanoparticles were shown to protect the content against cold storage, heat, high hydrostatic pressure processing, oxidation and ultraviolet (UV) radiation [40, 74, 77, 80, 82]. Nonetheless, reassembled casein nanoparticles are readily digested by gastrointestinal proteases [79–81]. This could be beneficial for stomach delivery, but when the intestine is targeted, additional factors should be introduced to reduce gastric digestion.

Interaction of caseins with other polymers was another process used for preparation of nanoparticles. Casein–pectin polyelectrolyte complexes were fabricated by slow acidification with glucono-δ-lactone and heating. Rutin was chosen as a model encapsulated compound. The obtained nanoparticles showed delayed proteolysis in simulated gastric conditions and sustained release in simulated intestinal conditions [84]. A casein–zein complex was used to co-encapsulate eugenol and thymol before spray-drying. After rehydration, the capsules were stable and allowed the content to exhibit bacteriostatic effects against *Escherichia coli* and *Listeria monocytogenes* [85]. Kessler et al. mixed casein rich in α_S_-casein with triblock copolymer PEO_13_–PPO_30_–PEO_13_ [13]. Micelles containing both polymers were formed and could solubilise pyrene.

Self-assembled nanoparticles were also prepared with chemically modified protein. Casein grafted with dextran was prepared by means of Maillard reaction [86]. β-Carotene was incorporated into spherical nanocapsules composed of a β-carotene core surrounded by casein molecules with dextran chains facing outside. The obtained nanocapsules were water soluble, stable, and upon digestion with proteases could release their content. Aqueous dispersion of nanoparticles was stable against oxidation, pH change and ionic strength change [86]. In other work, the Amadori rearrangement of the Maillard reaction was used to prepare casein-graft-dextran copolymer [87]. The product exhibited pH-dependent behaviour with micellisation occurring at pH equal to the pI of casein and successfully encapsulated pyrene as a model substance, used as a fluorescence marker. The Maillard reaction was also used to conjugate casein and maltodextrin [88]. Nanocapsules were able to protect vitamin D_2_ in gastric-like conditions (2 h, pH 2.5), but during simulated gastric digestion, Nile red (used as a hydrophobic substance model) was not released. The authors suggested potential for enteric delivery, even though such an approach was not tested.

Preparation of casein nanoparticles aided by application of cross-linking agents has been studied. Kumar and Singh prepared nanoparticles from casein and silk fibroin blend cross-linked with glutaraldehyde [89]. Particles were optimised using an in silico approach before preparation. Carvedilol in dimethylformamide was added to pre-fabricated nanoparticles, and after stirring and sonication the suspension was dialysed against water. The optimised formulation exhibited spherical shape and a twofold increase in maximum observed plasma concentration as well as 6.87 times increase in bioavailability compared with aqueous solution during in vivo experiments in rats. In another study, glutaraldehyde cross-linked casein was used to encapsulate magnetic particles [90]. Methanolic solution of doxorubicin was mixed with magnetic iron oxide nanoparticles coated with polymaleate and octadecene copolymer and incubated to allow the drug to be incorporated into the hydrophobic layer of the polymer. Particles were then coated with casein by deposition of casein molecules onto the particles followed by glutaraldehyde cross-linking. The casein-coated nanoparticles showed enhanced permeability compared with uncoated particles in ex vivo experiments. This phenomenon is thought to be attributable to energy-independent penetration of the plasma membrane by casein molecules [39, 90]. Additionally, the casein outer layer was shown to be resistant to pepsin at low pH and was digested by trypsin at neutral pH [90]. Clearly, glutaraldehyde cross-linking is responsible for the inhibition of gastric digestion of the otherwise readily digestible casein. This knowledge can be used to control digestion of nanocapsules targeted for the intestine. Even though glutaraldehyde is toxic [91], there are long established methods for determination of the smallest amounts of glutaraldehyde [92, 93], allowing monitoring of the reaction with proteins [92].

Glutaraldehyde can be substituted with non-toxic, or less toxic, cross-linking agents. Zhen et al. used transglutaminase to cross-link casein for preparation of nanoparticles loaded with cisplatin [94]. Nanoparticles enabled deep penetration into tumour tissue, and in mice with hepatic tumour growth, inhibition was better than with free cisplatin.

Cross-linked casein nanoparticles were recently used for preparation of swellable floating tablets [95]. Alfuzosin hydrochloride was dissolved directly in casein solution, and genipin in ethanol was added to achieve cross-linking. Nanoparticles were obtained by spray-drying [95, 96]. Powdered nanoparticles were then tableted using a single-punch tablet press. Tablets obtained by this method were compared with marketed alfuzosin formulation. Casein-based tablets floated much faster than marketed ones. Both formulations prolonged alfuzosin release for 24 h [95], whereas drug release could be modulated by altering the cross-linking degree in the case of the casein tablets [95, 96].

One of the most interesting papers describes nanoencapsulation in a freeze-concentrated (cryocentrated) phase [97]. This phase can be defined as a microspace with high concentration of ice crystals forming during freezing in aqueous solution. Casein and β-carotene were mixed and steadily frozen at −40 °C, then freeze-dried under vacuum at −20 °C. β-Carotene molecules were enclosed between the surface and interior of the nanoparticles. When aging under frozen conditions was introduced, the amount of β-carotene on the surface increased, along with the surface hydrophobicity. However, after rehydration, the surface hydrophobicity reverted.

### β-Casein nanoparticles

The ability to self-assemble is also observed for individual casein peptides such as β-casein, which forms globular micelles composed of 15–60 molecules with hydrodynamic radius of 7–14 nm [98, 99]. Extraction of β-casein involves three consecutive steps: separation of whole casein from milk, and isolation of β-casein; micelle disruption by acidification, chelation or cooling, and purification; washing, precipitation and separation [100].

β-Casein is found in micelles at pH above pI (5.33) and 15–30 °C, but is monomeric at 0–8 °C [7, 101], making these conformational changes temperature-driven characteristics. At 24 °C and pH 2.6, below pI, β-casein is present as round disk-like assemblies with width of 3–4 nm and diameter of 20–25 nm [102]. The CMC of β-casein is 0.3–0.7 mg/ml and depends on pH, temperature and ionic strength [99]. β-Casein molecules have charged hydrophilic N-terminal regions, whose electrostatic repulsion combined with the attraction of hydrophobic domains forms the basis of its micellisation (Fig. 2) [99]. The majority of the hydrophilic residues form the shell, while most of the hydrophobic residues form the core of β-casein micelles [103]. In fact, its behaviour resembles diblock copolymers [104].

In recent years, β-casein has been used for encapsulation of hydrophobic therapeuticals including celecoxib [105–107], ibuprofen [108], mitoxantrone [109, 110], naringenin [111], paclitaxel [112, 113], tariquidar [112] as well as *Vinca* alkaloids (vinblastine), taxanes (paclitaxel and docetaxel) and camptothecins (irinotecan) [101].

The β-casein used in the above works was of bovine origin, but casein produced by other species can also be used successfully. Interestingly, Esmaili et al. encapsulated curcumin in nanoparticles based on camel β-casein [114], although broader application may be limited as this encapsulation material is quite uncommon.

Similarly to whole casein, β-casein also offers protective abilities, in this case against lyophilisation [105].

## Conclusions

This review presents the perspectives for utilisation of caseins as encapsulation material. Caseins, being dietary proteins, are considered to be GRAS and are expected to be biocompatible, biodegradable and bioresorbable on oral administration. Their easy access and relatively high concentration in the raw material from which they are obtained, i.e. milk, make caseins inexpensive. Production of caseins is well known, long established and considered simple. Their flexible and unfolded conformation provides them with resistance to denaturation, which is beneficial during processing and manufacture.

The behaviour of casein micelles is driven by changes of pH, causing them to shrink in an acidic environment. This phenomenon is anticipated to confer protection of substances encapsulated in casein during passage through the harsh environment of the stomach. The open structure of caseins makes them vulnerable to proteolytic digestion, which in some cases can be beneficial, such as for complete release of encapsulated substances, but disadvantageous in others, e.g. unchecked burst release, but can be controlled by utilising cross-linking agents. This is probably due to rigidisation of protein structure and blocking of residues recognised by proteolytic enzymes. Caseins have also been shown to penetrate the plasma membrane in an energy-independent fashion, ensuring enhanced cellular uptake of encapsulated substances.

Casein has been used for both microencapsulation and nanoencapsulation of biologically active agents, including pharmaceuticals, probiotic cells, nutraceuticals and nutrients. Encapsulation approaches for pharmaceuticals differ from methods used for other substances, but this does not limit their potential for future work. Indeed, many approaches tested for nutraceuticals were eventually used for pharmaceuticals.

The remarkable gelation properties of casein have been used to prepare microcapsules that can encapsulate cells of probiotic bacteria. This process was facilitated by the enzymatic action of rennet or transglutaminase. However, additional work is necessary to improve the survival rate and stability of cells during storage. Much progress has also been made on coacervation of casein with polysaccharides to encapsulate hydrophobic substances. This process can be achieved by adjusting the pH to low values, as caseins gain positive charge in acidic pH, and can thus interact with the negative charge on polysaccharides. However, such methods are probably limited to acid-insensitive substances, as others may be damaged.

Caseins are naturally designed to bind and encapsulate high concentrations of calcium phosphate, via self-assembly into micelles. This, combined with the block distribution of hydrophobic regions of caseins, enables them to interact with lipophilic substances, providing a basis for nanoencapsulation of hydrophobic substances. In brief, particular factors are utilised to disrupt the micellar structure, hydrophobic substances are added and bind to caseins, followed by removal of disrupting factors and subsequent reassembly of caseins into micelles with the substances of interest entrapped inside. Such nanoparticles can provide solubilisation of lipophilic compounds in water-based solutions, in addition to improved bioavailability and protection against certain conditions. Recently, self-assembly was also studied for isolated β-casein. This biopolymer has a diblock structure in terms of charge and hydrophobicity distribution along the chain, providing the foundation for its spontaneous micellisation. From 0 to 30 °C, β-casein changes from monomeric to micellised. This temperature-driven process in such a mild thermal range suggests great potential for nanoencapsulation of sensitive compounds.

Different casein nanoparticles have been shown to protect their contents against cold (storage and lyophilisation), heat, oxidation, UV radiation, high hydrostatic pressure processing and changes of pH and ionic strength.

## References

[1] Beezer GR (1940). Latin and Greek roots in chemical terminology. J Chem Educ.

[2] Holt C, Carver JA, Ecroyd H, Thorn DC (2013). Invited review: caseins and the casein micelle: their biological functions, structures, and behavior in foods. J Dairy Sci.

[3] Artym J, Zimecki M (2013). Milk-derived proteins and peptides in clinical trials. Postȩpy Hig Med Dośw Online.

[4] Pereira PC (2014). Milk nutritional composition and its role in human health. Nutrition.

[5] Elzoghby AO, Abo El-Fotoh WS, Elgindy NA (2011). Casein-based formulations as promising controlled release drug delivery systems. J Control Release.

[6] Caroli AM, Chessa S, Erhardt GJ (2009). Invited review: milk protein polymorphisms in cattle: effect on animal breeding and human nutrition. J Dairy Sci.

[7] Abd El-Salam MH, El-Shibiny S (2012). Formation and potential uses of milk proteins as nano delivery vehicles for nutraceuticals: a review. Int J Dairy Technol.

[8] Farrell HM, Malin EL, Brown EM, Qi PX (2006). Casein micelle structure: what can be learned from milk synthesis and structural biology?. Curr Opin Colloid Interface Sci.

[9] Livney YD (2010). Milk proteins as vehicles for bioactives. Curr Opin Colloid Interface Sci.

[10] Audic J-L, Chaufer B, Daufin G (2003). Non-food applications of milk components and dairy co-products: a review. Le Lait.

[11] Fox PF, Brodkorb A (2008). The casein micelle: historical aspects, current concepts and significance. Int Dairy J.

[12] Swaisgood HE, Fox PF, McSweeney PLH (2003). Chemistry of the caseins. Advanced dairy chemistry proteins.

[13] Kessler A, Menéndez-Aguirre O, Hinrichs J (2014). α_s_-Casein—PE6400 mixtures: surface properties, miscibility and self-assembly. Colloids Surf B Biointerfaces.

[14] de Kruif CG, Huppertz T, Urban VS, Petukhov AV (2012). Casein micelles and their internal structure. Adv Colloid Interface Sci.

[15] Trejo R, Dokland T, Jurat-Fuentes J, Harte F (2011). Cryo-transmission electron tomography of native casein micelles from bovine milk. J Dairy Sci.

[16] Bouchoux A, Gésan-Guiziou G, Pérez J, Cabane B (2010). How to squeeze a sponge: casein micelles under osmotic stress, a SAXS study. Biophys J.

[17] CSN1S1–Alpha-S1-casein precursor–Bos taurus (Bovine)—CSN1S1 gene and protein. http://www.uniprot.org/uniprot/P02662. Accessed 10 Feb 2017

[18] CSN1S2–Alpha-S2-casein precursor–Bos taurus (Bovine)—CSN1S2 gene and protein. http://www.uniprot.org/uniprot/P02663. Accessed 10 Feb 2017

[19] CSN2–Beta-casein precursor–Bos taurus (Bovine)—CSN2 gene and protein. http://www.uniprot.org/uniprot/P02666. Accessed 10 Feb 2017

[20] CSN3–Kappa-casein precursor–Bos taurus (Bovine)—CSN3 gene and protein. http://www.uniprot.org/uniprot/P02668. Accessed 10 Feb 2017

[21] De Sa Peixoto P, Silva JVC, Laurent G (2017). How high concentrations of proteins stabilize the amorphous state of calcium orthophosphate: a solid-state nuclear magnetic resonance (NMR) study of the casein case. Langmuir.

[22] Gonzalez-Jordan A, Thomar P, Nicolai T, Dittmer J (2015). The effect of pH on the structure and phosphate mobility of casein micelles in aqueous solution. Food Hydrocoll.

[23] Tagliabracci VS, Engel JL, Wen J (2012). Secreted kinase phosphorylates extracellular proteins that regulate biomineralization. Science.

[24] Gebhardt R, Vendrely C, Kulozik U (2011). Structural characterization of casein micelles: shape changes during film formation. J Phys Condens Matter.

[25] Jollès P (1979). The carbohydrate portions of milk glycoproteins. J Dairy Res.

[26] Dalgleish DG (1998). Casein micelles as colloids: surface structures and stabilities. J Dairy Sci.

[27] de Kruif CG, Holt C, Fox PF, McSweeney PLH (2003). Casein micelle structure, functions and interactions. Advanced dairy chemistry proteins.

[28] Bauman DE, Mather IH, Wall RJ, Lock AL (2006). Major advances associated with the biosynthesis of milk. J Dairy Sci.

[29] Liu Y, Guo R (2008). pH-dependent structures and properties of casein micelles. Biophys Chem.

[30] Liu Z, Juliano P, Williams RP (2014). Ultrasound effects on the assembly of casein micelles in reconstituted skim milk. J Dairy Res.

[31] Tuinier R, de Kruif CG (2002). Stability of casein micelles in milk. J Chem Phys.

[32] Suárez-Luque S, Mato I, Huidobro JF, Simal-Lozano J (2007). Determination of major metal cations in milk by capillary zone electrophoresis. Int Dairy J.

[33] Ye R, Harte F (2013). Casein maps: effect of ethanol, pH, temperature, and CaCl_2_ on the particle size of reconstituted casein micelles. J Dairy Sci.

[34] Thomar P, Nicolai T, Benyahia L, Durand D (2013). Comparative study of the rheology and the structure of sodium and calcium caseinate solutions. Int Dairy J.

[35] Thomar P, Benyahia L, Durand D, Nicolai T (2014). The influence of adding monovalent salt on the rheology of concentrated sodium caseinate suspensions and the solubility of calcium caseinate. Int Dairy J.

[36] Philippe M, Le Graët Y, Gaucheron F (2005). The effects of different cations on the physicochemical characteristics of casein micelles. Food Chem.

[37] Salque M, Bogucki PI, Pyzel J (2013). Earliest evidence for cheese making in the sixth millennium bc in northern Europe. Nature.

[38] Quigley EMM, Turnberg LA (1987). pH of the microclimate lining human gastric and duodenal mucosa in vivo. Gastroenterology.

[39] Liu C, Yao W, Zhang L (2010). Cell-penetrating hollow spheres based on milk protein. Chem Commun.

[40] Semo E, Kesselman E, Danino D, Livney YD (2007). Casein micelle as a natural nano-capsular vehicle for nutraceuticals. Food Hydrocoll.

[41] Peixoto PDS, Bouchoux A, Huet S (2015). Diffusion and partitioning of macromolecules in casein microgels: evidence for size-dependent attractive interactions in a dense protein system. Langmuir.

[42] Vert M, Doi Y, Hellwich K-H (2012). Terminology for biorelated polymers and applications (IUPAC recommendations 2012). Pure Appl Chem.

[43] Chen Y, Willmott N, Anderson J, Florence AT (1987). Comparison of albumin and casein microspheres as a carrier for doxorubicin. J Pharm Pharmacol.

[44] Desoize B, Jardillier JC, Kanoun K (1986). In-vitro cytotoxic activity of cross-linked protein microcapsules. J Pharm Pharmacol.

[45] Burgain J, Gaiani C, Cailliez-Grimal C (2013). Encapsulation of *Lactobacillus rhamnosus* GG in microparticles: influence of casein to whey protein ratio on bacterial survival during digestion. Innov Food Sci Emerg Technol.

[46] Heidebach T, Först P, Kulozik U (2009). Transglutaminase-induced caseinate gelation for the microencapsulation of probiotic cells. Int Dairy J.

[47] Heidebach T, Först P, Kulozik U (2010). Influence of casein-based microencapsulation on freeze-drying and storage of probiotic cells. J Food Eng.

[48] Würth R, Hörmannsperger G, Wilke J (2015). Protective effect of milk protein based microencapsulation on bacterial survival in simulated gastric juice versus the murine gastrointestinal system. J Funct Foods.

[49] Wine Y, Cohen-Hadar N, Freeman A, Frolow F (2007). Elucidation of the mechanism and end products of glutaraldehyde crosslinking reaction by X-ray structure analysis. Biotechnol Bioeng.

[50] Griffin M, Casadio R, Bergamini CM (2002). Transglutaminases: nature’s biological glues. Biochem J.

[51] Butler MF, Ng Y-F, Pudney PDA (2003). Mechanism and kinetics of the crosslinking reaction between biopolymers containing primary amine groups and genipin. J Polym Sci Part Polym Chem.

[52] Guerin J, Petit J, Burgain J (2017). *Lactobacillus rhamnosus* GG encapsulation by spray-drying: milk proteins clotting control to produce innovative matrices. J Food Eng.

[53] Jain A, Thakur D, Ghoshal G (2016). Characterization of microcapsulated β-carotene formed by complex coacervation using casein and gum tragacanth. Int J Biol Macromol.

[54] Jain A, Thakur D, Ghoshal G (2016). Formation and functional attributes of electrostatic complexes involving casein and anionic polysaccharides: an approach to enhance oral absorption of lycopene in rats in vivo. Int J Biol Macromol.

[55] Koupantsis T, Pavlidou E, Paraskevopoulou A (2014). Flavour encapsulation in milk proteins–CMC coacervate-type complexes. Food Hydrocoll.

[56] Koupantsis T, Pavlidou E, Paraskevopoulou A (2016). Glycerol and tannic acid as applied in the preparation of milk proteins–CMC complex coavervates for flavour encapsulation. Food Hydrocoll.

[57] Marreto RN, Ramos MFS, Silva EJ (2013). Impact of cross-linking and drying method on drug delivery performance of casein–pectin microparticles. AAPS PharmSciTech.

[58] Matalanis A, Decker EA, McClements DJ (2012). Inhibition of lipid oxidation by encapsulation of emulsion droplets within hydrogel microspheres. Food Chem.

[59] Chen F, Liang L, Zhang Z (2017). Inhibition of lipid oxidation in nanoemulsions and filled microgels fortified with omega-3 fatty acids using casein as a natural antioxidant. Food Hydrocoll.

[60] Goyal A, Sharma V, Sihag MK (2015). Development and physico-chemical characterization of microencapsulated flaxseed oil powder: a functional ingredient for omega-3 fortification. Powder Technol.

[61] Mounsey JS, O’Kennedy BT, Kelly PM (2005). Comparison of re-micellised casein prepared from acid casein with micellar casein prepared by membrane filtration. Le Lait.

[62] Chu B-S, Ichikawa S, Kanafusa S, Nakajima M (2007). Preparation and characterization of β-carotene nanodispersions prepared by solvent displacement technique. J Agric Food Chem.

[63] Chu B-S, Ichikawa S, Kanafusa S, Nakajima M (2007). Preparation of protein-stabilized β-carotene nanodispersions by emulsification–evaporation method. J Am Oil Chem Soc.

[64] Ghasemi S, Abbasi S (2014). Formation of natural casein micelle nanocapsule by means of pH changes and ultrasound. Food Hydrocoll.

[65] Roach A, Dunlap J, Harte F (2009). Association of triclosan to casein proteins through solvent-mediated high-pressure homogenization. J Food Sci.

[66] Huppertz T, Grosman S, Fox PF, Kelly AL (2004). Heat and ethanol stabilities of high-pressure-treated bovine milk. Int Dairy J.

[67] Elzoghby AO, Helmy MW, Samy WM, Elgindy NA (2013). Micellar delivery of flutamide via milk protein nanovehicles enhances its anti-tumor efficacy in androgen-dependent prostate cancer rat model. Pharm Res.

[68] Elzoghby AO, Helmy MW, Samy WM, Elgindy NA (2013). Spray-dried casein-based micelles as a vehicle for solubilization and controlled delivery of flutamide: formulation, characterization, and in vivo pharmacokinetics. Eur J Pharm Biopharm.

[69] Pan K, Zhong Q, Baek SJ (2013). Enhanced dispersibility and bioactivity of curcumin by encapsulation in casein nanocapsules. J Agric Food Chem.

[70] Pan K, Luo Y, Gan Y (2014). pH-driven encapsulation of curcumin in self-assembled casein nanoparticles for enhanced dispersibility and bioactivity. Soft Matter.

[71] Yazdi SR, Bonomi F, Iametti S (2013). Binding of curcumin to milk proteins increases after static high pressure treatment of skim milk. J Dairy Res.

[72] Menéndez-Aguirre O, Stuetz W, Grune T (2011). High pressure-assisted encapsulation of vitamin D_2_ in reassembled casein micelles. High Press Res.

[73] Menéndez-Aguirre O, Kessler A, Stuetz W (2014). Increased loading of vitamin D_2_ in reassembled casein micelles with temperature-modulated high pressure treatment. Food Res Int.

[74] Haham M, Ish-Shalom S, Nodelman M (2012). Stability and bioavailability of vitamin D nanoencapsulated in casein micelles. Food Funct.

[75] Levinson Y, Ish-Shalom S, Segal E, Livney YD (2016). Bioavailability, rheology and sensory evaluation of fat-free yogurt enriched with VD_3_ encapsulated in re-assembled casein micelles. Food Funct.

[76] Cohen Y, Ish-Shalom S, Segal E (2017). The bioavailability of vitamin D_3_, a model hydrophobic nutraceutical, in casein micelles, as model protein nanoparticles: human clinical trial results. J Funct Foods.

[77] Sáiz-Abajo M-J, González-Ferrero C, Moreno-Ruiz A (2013). Thermal protection of β-carotene in re-assembled casein micelles during different processing technologies applied in food industry. Food Chem.

[78] Jarunglumlert T, Nakagawa K, Adachi S (2015). Influence of aggregate structure of casein on the encapsulation efficiency of β-carotene entrapped via hydrophobic interaction. Food Struct.

[79] Yi J, Lam TI, Yokoyama W (2015). Beta-carotene encapsulated in food protein nanoparticles reduces peroxyl radical oxidation in Caco-2 cells. Food Hydrocoll.

[80] Zhang Y, He S, Ma Y (2015). Characterization and bioaccessibility of β-carotene in re-assembled casein. RSC Adv.

[81] Penalva R, Esparza I, Agüeros M (2015). Casein nanoparticles as carriers for the oral delivery of folic acid. Food Hydrocoll.

[82] Zimet P, Rosenberg D, Livney YD (2011). Re-assembled casein micelles and casein nanoparticles as nano-vehicles for ω-3 polyunsaturated fatty acids. Food Hydrocoll.

[83] Koo SY, Mok I-K, Pan C-H, Kim SM (2016). Preparation of fucoxanthin-loaded nanoparticles composed of casein and chitosan with improved fucoxanthin bioavailability. J Agric Food Chem.

[84] Luo Y, Pan K, Zhong Q (2015). Casein/pectin nanocomplexes as potential oral delivery vehicles. Int J Pharm.

[85] Chen H, Zhang Y, Zhong Q (2015). Physical and antimicrobial properties of spray-dried zein–casein nanocapsules with co-encapsulated eugenol and thymol. J Food Eng.

[86] Pan X, Yao P, Jiang M (2007). Simultaneous nanoparticle formation and encapsulation driven by hydrophobic interaction of casein-graft-dextran and β-carotene. J Colloid Interface Sci.

[87] Pan X, Mu M, Hu B (2006). Micellization of casein-graft-dextran copolymer prepared through Maillard reaction. Biopolymers.

[88] Markman G, Livney YD (2012). Maillard-conjugate based core-shell co-assemblies for nanoencapsulation of hydrophobic nutraceuticals in clear beverages. Food Funct.

[89] Kumar S, Singh SK (2017). In silico–in vitro–in vivo studies of experimentally designed carvedilol loaded silk fibroin-casein nanoparticles using physiological based pharmacokinetic model. Int J Biol Macromol.

[90] Huang J, Shu Q, Wang L (2015). Layer-by-layer assembled milk protein coated magnetic nanoparticle enabled oral drug delivery with high stability in stomach and enzyme-responsive release in small intestine. Biomaterials.

[91] Zeiger E, Gollapudi B, Spencer P (2005). Genetic toxicity and carcinogenicity studies of glutaraldehyde—a review. Mutat Res Mutat Res.

[92] Boratyński J, Żal T (1990). Colorimetric micromethods for glutaraldehyde determination by means of phenol and sulfuric acid or phenol and perchloric acid. Anal Biochem.

[93] Sawicki E, Hauser TR, Stanley TW, Elbert W (1961). The 3-methyl-2-benzothiazolone hydrazone test. Sensitive new methods for the detection, rapid estimation, and determination of aliphatic aldehydes. Anal Chem.

[94] Zhen X, Wang X, Xie C (2013). Cellular uptake, antitumor response and tumor penetration of cisplatin-loaded milk protein nanoparticles. Biomaterials.

[95] Elzoghby AO, Vranic BZ, Samy WM, Elgindy NA (2015). Swellable floating tablet based on spray-dried casein nanoparticles: near-infrared spectral characterization and floating matrix evaluation. Int J Pharm.

[96] Elzoghby AO, Samy WM, Elgindy NA (2013). Novel spray-dried genipin-crosslinked casein nanoparticles for prolonged release of alfuzosin hydrochloride. Pharm Res.

[97] Nakagawa K, Jarunglumlert T, Adachi S (2016). Structural changes in casein aggregates under frozen conditions affect the entrapment of hydrophobic materials and the digestibility of aggregates. Chem Eng Sci.

[98] Portnaya I, Cogan U, Livney YD (2006). Micellization of bovine β-casein studied by isothermal titration microcalorimetry and cryogenic transmission electron microscopy. J Agric Food Chem.

[99] Faizullin DA, Konnova TA, Haertlé T, Zuev YF (2017). Secondary structure and colloidal stability of beta-casein in microheterogeneous water-ethanol solutions. Food Hydrocoll.

[100] Atamer Z, Post AE, Schubert T (2017). Bovine β-casein: isolation, properties and functionality. A review. Int Dairy J.

[101] Shapira A, Assaraf YG, Epstein D, Livney YD (2010). Beta-casein nanoparticles as an oral delivery system for chemotherapeutic drugs: impact of drug structure and properties on co-assembly. Pharm Res.

[102] Portnaya I, Ben-Shoshan E, Cogan U (2008). Self-assembly of bovine β-casein below the isoelectric pH. J Agric Food Chem.

[103] de Kruif CG, Grinberg VY (2002). Micellisation of β-casein. Colloids Surf Physicochem Eng Asp.

[104] Horne DS (2002). Casein structure, self-assembly and gelation. Curr Opin Colloid Interface Sci.

[105] Bachar M, Mandelbaum A, Portnaya I (2012). Development and characterization of a novel drug nanocarrier for oral delivery, based on self-assembled β-casein micelles. J Control Release.

[106] Perlstein H, Turovsky T, Gimeson P (2015). Thermotropic behavior of celecoxib-loaded beta-casein micelles: relevance to the improved bioavailability. Eur J Nanomed.

[107] Turovsky T, Khalfin R, Kababya S (2015). Celecoxib encapsulation in β-casein micelles: structure, interactions, and conformation. Langmuir.

[108] Turovsky T, Portnaya I, Kesselman E (2015). Effect of temperature and loading on the structure of β-casein/ibuprofen assemblies. J Colloid Interface Sci.

[109] Shapira A, Assaraf YG, Livney YD (2010). Beta-casein nanovehicles for oral delivery of chemotherapeutic drugs. Nanomed Nanotechnol Biol Med.

[110] Shapira A, Markman G, Assaraf YG, Livney YD (2010). β-Casein-based nanovehicles for oral delivery of chemotherapeutic drugs: drug-protein interactions and mitoxantrone loading capacity. Nanomed Nanotechnol Biol Med.

[111] Moeiniafshari A-A, Zarrabi A, Bordbar A-K (2015). Exploring the interaction of naringenin with bovine beta-casein nanoparticles using spectroscopy. Food Hydrocoll.

[112] Bar-Zeev M, Assaraf YG, Livney YD (2016). β-Casein nanovehicles for oral delivery of chemotherapeutic drug combinations overcoming p-glycoprotein-mediated multidrug resistance in human gastric cancer cells. Oncotarget.

[113] Shapira A, Davidson I, Avni N (2012). β-Casein nanoparticle-based oral drug delivery system for potential treatment of gastric carcinoma: stability, target-activated release and cytotoxicity. Eur J Pharm Biopharm.

[114] Esmaili M, Ghaffari SM, Moosavi-Movahedi Z (2011). Beta casein-micelle as a nano vehicle for solubility enhancement of curcumin; food industry application. LWT-Food Sci Technol.

